# Development and Bioanalytical Applications of a White Light Reflectance Spectroscopy Label-Free Sensing Platform

**DOI:** 10.3390/bios7040046

**Published:** 2017-10-13

**Authors:** Georgios Koukouvinos, Panagiota Petrou, Dimitrios Goustouridis, Konstantinos Misiakos, Sotirios Kakabakos, Ioannis Raptis

**Affiliations:** 1 Immunoassay/Immunosensors Lab, Institute of Nuclear & Radiological Sciences & Technology, Energy & Safety, NCSR “Demokritos”, 15310 Aghia Paraskevi, Greece; geokoukoubinos@yahoo.gr (G.K.); ypetrou@rrp.demokritos.gr (P.P.); skakab@rrp.demokritos.gr (S.K.); 2 ThetaMetrisis S.A., 12243 Egaleo, Greece; dgousto@teipir.gr; 3 Department of Electronics Engineering TEI of Piraeus, 12244 Egaleo, Greece; 4 Optical sensors Lab, Institute of Nanoscience and Nanotechnology, NCSR “Demokritos”, 15310 Aghia Paraskevi, Greece; k.misiakos@inn.demokritos.gr

**Keywords:** white light reflectance spectroscopy, label-free determinations, fast assays, volatile organic compounds, protein analytes, low molecular weight molecules

## Abstract

The development of a sensing platform based on white light reflectance spectroscopy (WLRS) is presented. The evolution of the system, from polymer film characterization and sensing of volatile organic compounds to biosensor for the label-free determination of either high (e.g., proteins) or low molecular weight analytes (e.g., pesticides), is described. At the same time, the passage from single to multi-analyte determinations, and from a laboratory prototype set-up to a compact device appropriate for on-site determination, is outlined. The improvements made on both the sensor and the optical set-up, and the concomitant advances in the analytical characteristics and the robustness of the assays performed with the different layouts, are also presented. Finally, the future perspectives of the system, aiming for the creation of a standalone instrument to be used by non-experts, will be discussed.

## 1. Introduction

Amongst the different types of sensors that can be incorporated to microfluidic systems in order to build up miniaturized systems for Point of Care (PoC) applications, the optical ones present the advantages of direct and multiplex analysis, due to lack of strong interference from the sample matrix that is the major limitation of electrochemical sensors [1,2,3]. Although the early optical sensors relied on the implementation of labels to convert biomolecular interactions into detectable and quantifiable signals, in the last decades, research effort has focused on label-free transduction principles [4,5]. Label-free sensors are able to produce a signal upon the binding of the analyte to the biorecognition molecule that has been immobilized onto their surface, thus enabling for real-time monitoring of the biomolecular reaction, kinetic measurements, and faster assays. Nowadays, the label-free optical transduction principles with the higher contribution to research reports and the greatest potential for commercialization, are based on surface plasmon resonance (SPR), interferometry (Mach–Zehnder, Young, bi-modal interferometers), reflectometric interference spectroscopy, and ring resonators. The optical label-free methods can be divided in two main categories, refractometric and reflectometric [6]. In refractometric transducers, the radiation waveguided by total internal reflection, senses the cover medium through the evanescent wave field, which exponentially decays into the layer close to the waveguide surface. The waveguide surface is biofunctionalized with recognition biomolecules that bind the analyte when the samples flows over the sensor surface, resulting in a thickness change of the adlayer over the sensor surface. This change in thickness influences the evanescent field and its coupling back into the waveguide, which is quantified as a reduction in the intensity or the reflected beam, depending on angle or wavelength. Refractometric transducers include SPR, grating couplers, resonant mirrors, Mach–Zehnder and Young interferometers, and Bragg gratings. Despite the fact that refractometric optical sensors are the most abundant, and also those that have reached the higher commercialization level, they can “sense” phenomena that take place only within the evanescent field, and with an efficiency that is reduced as the layer thickness increases. Moreover, their response is temperature dependent, and temperature control is essential for response stabilization. On the other hand, in reflectrometric sensors, the radiation is reflected by the different interfaces that compose the transducer, usually including the layer of the recognition biomolecules and a dielectric material, creating an interference spectrum. The analyte binding onto recognition biomolecules induces a layer thickness increase that affects the reflected beams, leading to a shift in the interference spectrum. In the reflectrometric sensors, the effective biomolecular layer can extend to several nanometers, and is less vulnerable to temperature fluctuations. The most common reflectrometric sensing method is the one introduced by Gauglitz et al. in 1991, known as reflectometric interference spectroscopy (RIfS) [7]. In RIfS, the sensing element is a glass slide modified with a thin layer of transparent dielectric material (e.g., SiO_2_, SiO_2_–Ta_2_O_5_) on top of which the biomolecular reactions take place. When the white light strikes the glass from the back side, the partial beams, which are reflected at each interface, interfere, creating a reflectance spectrum with alternating maxima and minima corresponding to constructive and destructive interference of the reflected radiation. The build-up of an adlayer on top of the dielectric, due to biomolecular reactions, increases the optical path length, resulting in a shift of the reflectance spectrum. This shift is analogous to the thickness increase, and can be correlated with the concentration of the reacting biomolecules. Over the years, the method has evolved from the implementation of white light source and recording of the whole reflection spectrum [8,9], to monitoring of few wavelengths for multiplex detection in microtiter plates or sensor arrays [10,11], and to single wavelength set-ups suitable for imaging [12]. In addition, other substrates have been exploited as sensor elements in reflectometric interference spectroscopy systems, including porous silicon [13,14,15,16,17], porous silicon with thermally grown oxide [18], porous silicon-C composites [19], or other porous materials, such as TiO_2_ [20]. Although the sensing systems based on reflectrometric interference are advantageous as compared to systems based on refractometry in terms of simplicity, robustness, and instrumentation cost, the cases of commercially available systems based on reflectrometric interference transduction principles that been successfully launched, is rather limited [21,22]. This fact is in contradiction with the proven high analytical performance in a variety of applications, with a sensitivity and accuracy that is similar, or superior to, established analytical techniques.

Nevertheless, the analytical performance is only one of the parameters that could facilitate the acceptance of a new sensing system and its application outside the laboratory environment where it has been developed. To this end, the simplicity of the measurement procedure plays an important role (ideally the user should only load the sample), the cost effectiveness both in terms of the non-disposable instrumentation required and the consumables, and the ability to work with complex matrices [5]. Having all these considerations in mind, the present article aims to review the development and analytical evaluation of a sensing platform developed by our group the last decade. The sensing platform is based on White Light Reflectance Spectroscopy (WLRS), that is also a reflectometric optical sensing method. In particular, we report the advancements of the specific sensing platform regarding its application to different fields as well as its evolution from a lab prototype to a compact device for use at the Point-of-Care.

## 2. Operating Principle

**Figure 1:**
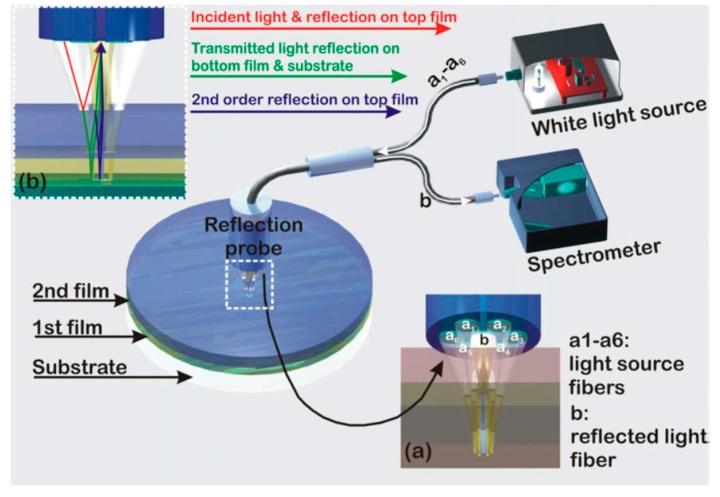
Typical white light reflectance spectroscopy (WLRS) set-up with detailed depiction of (**a**) reflection probe configuration, and (**b**) the light optical path through a two layer substrate. Reproduced with permission from Elsevier B.V. [13].

White light reflectance spectroscopy (WLRS) is based on the reflection of a broadband light beam (in visible (VIS) or in the visible/near infrared (VIS/NIR)) from an engineered surface, so that the reflected spectrum will have at least an interference fringe in the visible spectrum. Figure 1 schematically illustrates the principle of operation of the WLRS biosensor, as well as the basic components of the measurement set-up, i.e., the light source, the spectrometer, and the reflection probe [23]. The major differences with respect to typical RIfS sensors are the use of Si substrate instead of a transparent substrate, and the fact that the illumination and the collection of the reflected light is done through the fluidic cell, instead of the back of the transparent substrate. Despite the fact that the optical path of the light passes through the running liquid on top of the sensor, we have not experienced problems with air bubbles or turbid liquids, thanks to the design and dimensions of the microfluidic cell. The use of Si substrate allows also for the use of mainstream microelectronic processes, for the realization of large volumes of chips with identical properties at a very low cost. Furthermore, through the application of microelectronic processes, the number of sensing areas on the chip can be extended, and allow for multi-analyte monitoring in real-time without any moving components, and without increase of the cost and complexity of the measurement set-up. The reflection probe consists of a central fiber surrounded by another six, all with the same diameter. The six fibers (illumination) at the periphery of the probe deliver the light to the surface, whereas the central fiber collects the specular reflected light. The sensing element consists of a single layer or a stack of films from transparent materials with different refractive indices over a substrate of moderate reflectance, usually Si. As the light emitted from the light source is guided by the reflection probe vertically to this structure (single layer or stack of films on Si), it is reflected by the Si surface, and by the transparent materials layers of different refractive indexes. This way, interference take place at each wavelength, resulting in an interference spectrum that is collected by the central fiber of the reflection probe, and guided to the spectrometer where it is continuously recorded.

**Figure 2:**
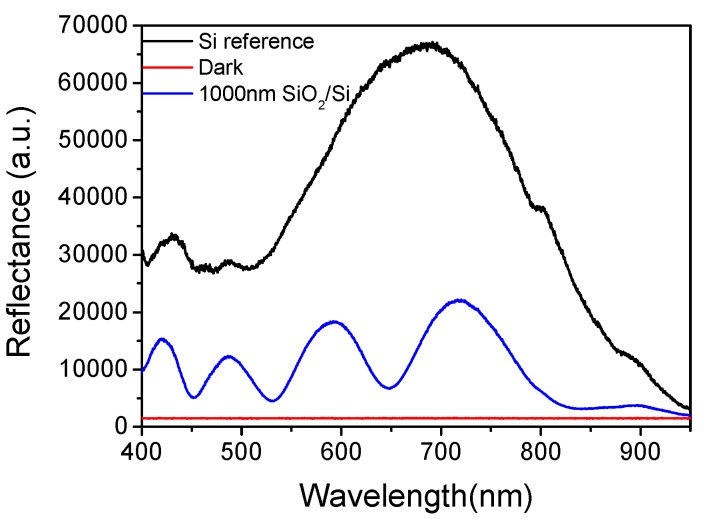
Typical reflectance spectrum from a Si surface with a 1000 nm SiO_2_ layer (blue line), and a plain Si surface (black line). The red line corresponds to dark spectrum, i.e., the reflectance spectrum from the plain Si surface obtained with the light source turned off.

In such a set-up, the total reflection coefficient for a surface consisting of k-layers can be calculated by various models. In our case, the Abeles approach was followed, where the effective Fresnel coefficient for the k − 1 layer is:

(1) $$\rho_{k-1^{e^{{i\Delta }_{\kappa -1}}}}=\frac{r_{k-1}+\rho_{k^{e^{{i\Delta }_{k}-2{i\delta }_{k-1}}}}}{1+r_{k-1}\rho_{k^{e^{{i\Delta }_{k}-2{i\delta }_{k}}}}}$$

where ρ is the amplitude, and Δ the phase. From this set of equations, the reflectance from any layer can be calculated. In the particular case of the present study, i.e., two transparent layers between the substrate and the environment, the total energy can be written as

(2) $$E=\frac{A}{B}$$

(3) $$\begin{array}{c} A=r_{01}^{2}+r_{12}^{2}+r_{23}^{2}+2r_{01}r_{12}^{2}r_{23}^{2}+r_{01}^{2}r_{12}^{2}r_{23}^{2}+2r_{01}r_{23}\cos\left(\frac{4\pi }{\lambda }\left(n_{1}d_{1}+n_{2}d_{2}\right)\right)\\ \;+2r_{12}r_{23}\cos\left(\frac{4\pi }{\lambda }n_{1}d_{1}\right)+2r_{01}r_{12}r_{23}^{2}\cos\left(\frac{4\pi }{\lambda }n_{2}d_{2}\right)\\ \;+2r_{01}r_{12}\cos\left(\frac{4\pi }{\lambda }n_{1}d_{1}\right)+2r_{01}^{2}r_{12}r_{23}\cos\left(\frac{4\pi }{\lambda }n_{1}d_{1}\right)\\ B\;=\;1+r_{01}^{2}r_{12}^{2}+r_{01}^{2}r_{23}^{2}+r_{12}^{2}r_{23}^{2}+2r_{12}r_{23}\cos\left(\frac{4\pi }{\lambda }n_{2}d_{2}\right)\\ \;+2r_{01}r_{23}\cos\left(\frac{4\pi }{\lambda }\left(n_{1}d_{1}+n_{2}d_{2}\right)\right)+2r_{01}^{2}r_{12}r_{23}\cos\left(\frac{4\pi }{\lambda }n_{2}d_{2}\right)\\ \;+2r_{01}r_{12}\cos\left(\frac{4\pi }{\lambda }n_{1}d_{1}\right)+2r_{01}r_{12}r_{23}^{2}\cos\left(\frac{4\pi }{\lambda }n_{1}d_{1}\right)\\ \;+2r_{01}r_{12}^{2}r_{23}\cos\left(\frac{4\pi }{\lambda }(n_{1}d_{1}+n_{2}d_{2})\right) \end{array}$$

where n_i_ is the refractive index of the *i*th layer (i = 0 for air, 1 for the first film, 2 for the second film and 3 for the substrate, and in the case of suspending film, stack n_3_ corresponds to air), and d_i_ of the *i*th layer, and λ the corresponding wavelength. The light propagation at the various interfaces is illustrated in Figure 1. A typical reflectance spectrum (arb. units) for a 1000 nm thick SiO_2_ layer on a Si substrate is illustrated in Figure 2, along with the reference spectrum from a plain Si substrate. The referencing is quite a critical step for the fitting of the reflectance spectrum with the interference equation, and the measurement of the minute spectral shifts due to bioreactions. The reference is collected by a Si chip that is placed in the docking station. Due to the high stability of the light source, the reference should be recorded sporadically i.e., once a week.

The number and the shape of interference fringes depends on the thickness and the refractive index of the transparent film(s). The fitting of the experimental reflectance spectrum with the above equation is performed using the Levenberg–Marquardt algorithm. By applying Equation (3) for all wavelengths in the desirable spectral range, the film thickness is calculated accurately and very fast, fulfilling the requirement for real-time monitoring of the bioreactions taking place on the surface of the biofunctionalized transducer. Through this procedure, the thickness of thin and ultrathin films with minimum detectable thickness change in the sub-Angstrom region has been demonstrated. The WLRS platform has been applied to determine the thermal behavior of polymeric layers deposited on Si substrates, the content of vapor mixtures in volatile organic compounds, and the real-time monitoring of biomolecular reactions.

## 3. System Development and Applications

The WLRS system was first introduced as a tool to determine the thickness of polymeric films deposited or spin-coated onto substrates during exposure to solvents [24] or thermal treatment [25], aiming to determine the dissolution properties or glass transition temperature, respectively, of these materials. The method proved to provide more consistent data when compared with two standard optical methods for film characterization, namely single-wavelength interferometry and spectroscopic ellipsometry. The changes in the polymer films’ thickness were determined by recording the reflected interference spectrum shift in the course of the experiment. Thus, the implementation of the same principle, and more or less of the same optical set-up for gas sensing applications, was the natural course of things.

### 3.1. Application to Sensors for Detection of Volatile Organic Compounds

The first application of WLRS as a sensing platform was for the quantitative detection of volatile organic compounds (VOC) through monitoring of polymeric films thickness changes due to exposure to VOCs [26,27,28]. Polymeric films tend to swell in the presence of gases with good affinity. The swelling of the polymeric film can be measured by reflectrometric methods, and then exploited for the measurement of the concentration of the gas. This approach can be expanded in the infrared spectrum and provide an infrared (IR) fingerprint of the gases, in addition to the measurement of the gas concentration [29], at the expense of larger and more expensive set-ups. In that first WLRS sensor configuration, the light from the VIS/NIR source was equally divided into two beams: one directed to the slave channel of a double-channel spectrophotometer, and another butt-coupled to the illumination fiber of the reflection probe. The sensor was a Si wafer with a thermally grown SiO_2_ layer that produced interference fringes within the spectral range of the spectrometer (see Figure 2). This reflectance spectrum was collected by the central fiber of the reflection probe, and guided to the master channel of the spectrophotometer. Both the sensor element and the reflection probe were placed in a chamber where controlled mixtures of VOC (e.g., methanol, ethanol, and acetone) with nitrogen were continuously flows. The polymers examined included mainly poly(hydroxy ethyl methacrylate) (PHEMA) and poly (methyl methacrylate) (PMMA), but also poly(isobutyl methacrylate) (PIBMA), poly(dimethylsiloxane-co-diphenylsiloxane) divinyl terminated (P(DMS-co-DPhS)-1), poly(dimethylsiloxane-co-diphenylsiloxane) dihydroxy terminated (P(DMS-co-DPhS), poly(3-butyltiophene-2,5-diyl)/polystyrene blends (PT/PS), as well as a variety of poly(3-alkylthiophene)s (P3ATs). PHEMA [26] was the first material tested with respect to its swelling behavior towards methanol and ethanol vapors at concentrations as high as 20,000 ppm. It was found that the thinner films exhibited higher relative expansion, with the 55 nm thick film providing the shorter response time and the highest calibration curve sensitivity. The study was expanded to PMMA films, leading to similar results [27,28] with respect to response to VOCs as a function of the film thickness. Then, the method was applied to evaluate the vapor sorption properties of homologous series of polymers, such as poly(alkyl methacrylates), poly(2-hydroxyethyl methacrylate) and poly(dimethylsiloxane-co- diphenylsiloxane)copolymers against water, methanol, ethanol and ethyl acetate vapors [30], as well as of poly(3-alkylthiophene)s against chloroform, tetrahydrofuran, cyclohexanone, and water [31]. Apart from polymers and co-polymers, the method was applied to study the sorption behavior against different VOCs of polymer blends consisting of two polymers with distinct solubility properties in water and organic solvents. These polymers acquired different film morphologies depending on the solvent employed for their spin-coating onto the Si surface [32]. The system was able to continuously monitor the interference spectrum and convert the observed spectral shifts to film thickness changes, through a dedicated software, thus enabling the recording of VOCs sorption and desorption curves in real-time, and their correlation with the VOC concentration in the gas by employing either the equilibrium signal or kinetic determination based on the initial sorption rate. Those studies proved the indisputable validity of the system developed to characterize polymer film properties, a feature that was further exploited in the frame of WLRS methodology commercialization by ThetaMetrisis, but also its ability to perform as gas sensor.

### 3.2. Application to Biomolecular Reaction Monitoring

**Figure 3:**
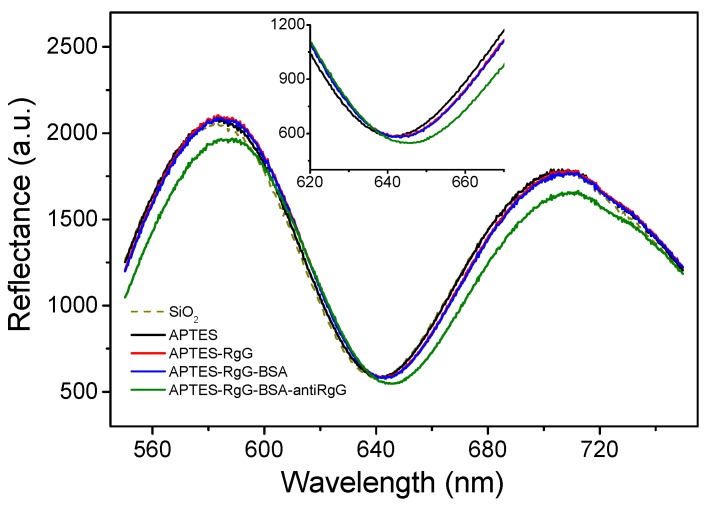
Shifts in reflectance interference spectrum of a Si/SiO_2_ substrate after chemical activation with APTES, adsorption of rabbit IgG, blocking with BSA, and reaction with anti-rabbit IgG antibody. The insert shows a blow-up of the wavelength range around the interference minimum peak.

The implementation of WLRS technology in biosensing applications was initiated by the determination of the thickness of layers created on Si-based surfaces after chemical activation, biofunctionalization, and biomolecular binding reactions [23]. The set-up used was similar to that employed to monitor the swelling of polymer films (Figure 1). Si wafers with a 1000 nm thick SiO_2_ layer were chemically activated with 3-aminopropyl(triethoxysilane) (APTES), to facilitate the immobilization of rabbit or mouse γ-globulins through physical adsorption. Then, the surface free binding sites were blocked through incubation with a bovine serum albumin solution, and the immobilized surface molecules were reacted with a goat anti-rabbit or anti-mouse IgG antibody, respectively. After each incubation step, the surfaces were washed with distilled water to remove the excess of reagents, and dried prior to acquisition of interference spectrum (Figure 3). The effect of probe concentration and incubation duration with the surfaces were studied, and optimum conditions were defined based on WLRS results.

**Figure 4:**
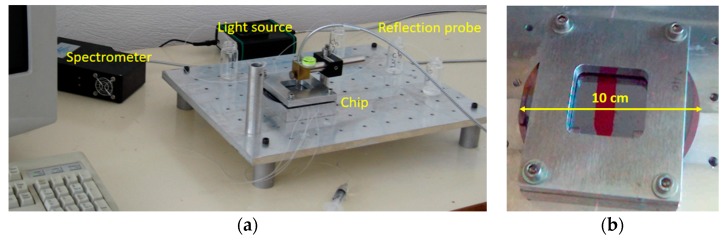
Photos of the first WLRS biosensing system. (**a**) Overview of the whole system. (**b**) The fluidic module with the 4-inch Si/SiO_2_ wafer. Reproduced with permission from Elsevier B.V.

The thickness values measured by WLRS were in good agreement with those determined for the same surfaces after processing of AFM images, providing further proof of the measurement accuracy in ultra-thin layers [23]. In particular, the thicknesses determined for the APTES layer by AFM and WLRS were 1.00 and 1.29 nm, respectively, and were increased after adsorption of rabbit IgG to 2.85 and 2.70 nm, respectively, and finally to 5.90 and 6.30 nm, respectively, after blocking of the surface and reaction with a goat anti-rabbit IgG antibody. These measurements were the proof of concept that the WLRS system could be used to “monitor” biomolecular interactions. The real-time monitoring, however, could be explored only by combination of the WRLS set-up with a fluidic compartment. The first attempt towards this direction is depicted in the photos of Figure 4. The fluidic channel was formed on a polymerized polydimethylsiloxane (PDMS) sheet with embedded inlet and outlet tubing which was applied on a 4 inch Si wafer that was modified with a 1000 nm thick SiO_2_ layer. On top of this, a 40 nm layer photoresist film (AZ-5214; AZ-EM materials) was spin-coated and post-baked at a high temperature (180 °C, 60 min) to create a very robust film that served as an alternative to chemical activation of the Si/SiO_2_ wafer with APTES, providing for protein immobilization through physical adsorption. The fluidic chamber was closed by a glass which provided a transparent window for the optical probing of the surface, and was fastened to the fluidic and the Si/SiO_2_/AZ wafer by means of an aluminum holder (Figure 4a), forming a reaction chamber with a volume of 0.5 mL. The reflectance spectrum was processed as presented above, while variations in the probing light spectral content caused by light source drifts or by optical fiber bending and relaxations, could be compensated by monitoring the reference spectrum. Nevertheless, in order to avoid even small differences between the spectral content of the probing and the reference beam, fitting was restricted in a short spectral region around the main interference peak (l_max_). Thus, taking into account that the refractive indices were for the protein layer n_0_ = 1.46, for the SiO_2_-photoresist layer n_1_ = 1.40, and for Si n_3_ = 4.00, the formula for the calculation of the protein’s layer thickness increase (Dd_1_) simplifies to Dd_1_/d_2_ = 2.06 Dl/l, where D_l_ is the l_max_ shift, and d_1_ and d_2_ are the thicknesses of the protein and the composite SiO_2_-photoresist layer, respectively.

The described set-up and signal processing approach was used at first to monitor in real-time the protein film build-up during immobilization of biotinylated bovine serum albumin (BSA) onto the sensor surface, blocking of the surface free binding sites with excess of BSA and specific interaction of immobilized biotin moieties with streptavidin [33]. The interaction of biotin with avidin or streptavidin is one of the most popular model binding assays employed in preliminary evaluation of sensors [34]. In order to evaluate the ability of the system for regeneration and reuse of the biomolecule-coated chips, another model binding assay was implemented: that of mouse γ-globulins binding onto immobilized anti-mouse IgG antibody [35,36]. Mouse γ-globulins concentrations ranging from 0.1 to 100 nM were tested, and calibration curves were constructed using either the endpoint signal after 10 min of reaction, or by calculating the reaction rate at the first minute of the reaction. Both approaches provided a detection limit of 150 pM, showing that by implication of initial reaction rate, the assay time could be considerably suppressed. Moreover, the regeneration of the immobilized antibody was tested, and the use of the same chip for up to seven times without loss of the antibody binding activity was demonstrated [36]. In addition, a simplification of data processing was also introduced with recording the reference spectrum only once at the beginning of the measurement. This enabled the use a single-channel spectrometer for monitoring the bioreactions, with pronounced effect on the cost and simplicity of the measuring apparatus. More specifically, both a reference (REF(λ)) and a dark spectrum (D(λ)) were acquired prior to start the continuous recording of the reflectance spectrum (S(λ)) (Figure 2), and the absolute reflectance spectrum was calculated by the Equation (4):

(4) $$R\left(\lambda \right)=\frac{S\left(\lambda \right)-D\left(\lambda \right)}{\mathrm{REF}\left(\lambda \right)-D\left(\lambda \right)}$$

This simplification of the experimental set-up was the first step towards the objective of a compact small size instrument appropriate for routine use outside the lab.

The same set-up was employed for the detection of single nucleotide polymorphisms (SNPs) related to breast cancer. In particular, one deleterious mutation in breast cancer 1 (BRCA1) gene, the 3099delT mutation, was selected, and a probe corresponding to the mutant sequence was immobilized onto the sensing chip [36]. In order to achieve oligonucleotide immobilization by physical adsorption, a protein/oligonucleotide conjugate was synthesized and used. Then, a complementary sequence probe was introduced at different concentrations in the reaction cell, and its interaction with the immobilized probe was monitored in real-time. The observed shift in the reflectance spectrum could be attributed, as in the case of antigen–antibody reactions, to thickness increase due to formation of double strand DNA. Nevertheless, other phenomena, such as refractive index change due to conformational changes of DNA strands upon hybridization, could have also contributed to the observable signal. The oligonucleotide concentrations tested fall within the range of PCR product concentration, demonstrating the ability of the sensor to monitor DNA hybridization reactions. As in the case of anti-mouse IgG antibody/mouse γ-globulin reactions, regeneration and reuse of the chip, as well as kinetic measurements, were possible.

The sensor was also applied for the quantitative determination of the complement activation product C3b in human serum samples [37]. C3b is generated during activation of the complement cascade as a result of enzymatic cleavage of complement component C3 into two fragments, C3a and C3b, and plays a central role in the initiation and amplification of the complement response. C3b attaches covalently to cell-surface antigens, leading to the opsonization of foreign cells, and stimulation of the adaptive immune response. In addition to that, C3 cleavage has been observed in several physiological as well as pathological conditions, including inflammatory, ischemic, and autoimmune diseases. Thus, the determination of C3d levels in the serum of patients with autoimmune diseases could help to evaluate the status of the disease and prevent acute events. For the detection of C3b with the WLRS sensor, a highly specific monoclonal antibody that could recognize C3b and its degradation products (iC3b, and C3c), but not intact C3, was used. The sensor evaluation was performed using either purified C3b solutions or dilutions of serum where complement was activated though treatment with zymosan A from *Saccharomyces cerevisiae*. In the latter case, the respective dilutions of plasma collected in EDTA-coated tubes were employed as negative control, since EDTA inhibits complement activation. Again, kinetic measurements were employed to suppress the assay time to 60 s, and the lowest detectable concentration determined was 20 ng/mL in terms of purified C3b, and a 6000-fold dilution in case of complement-activated serum samples. In addition, the dynamic range of the assay (0.02–2 μg/mL in terms of purified C3b and 1/3000–1/100 in terms of serum dilution) covered a significant range of clinically observed complement activation levels [38]. The analytical performance of the WLRS sensor was compared to that of an assay employing the same antibody, and adapted to a commercially available SPR instrument (Biacore X). As it was demonstrated, the SPR assay provided only two-fold lower detection limit compared to the WLRS-based sensor. Nevertheless, the comparison in terms of cost and size between of the two instruments clearly favors the WLRS set-up. It should be noted, that C3b is an emerging biomarker not yet used in every day clinical praxis. Nevertheless, the determination of its serum levels are widely used in research involving the development of new drugs targeting to complement system regulation, in order to effectively treat a broad spectrum of immune, inflammatory, or age-related diseases [38].

**Figure 5:**
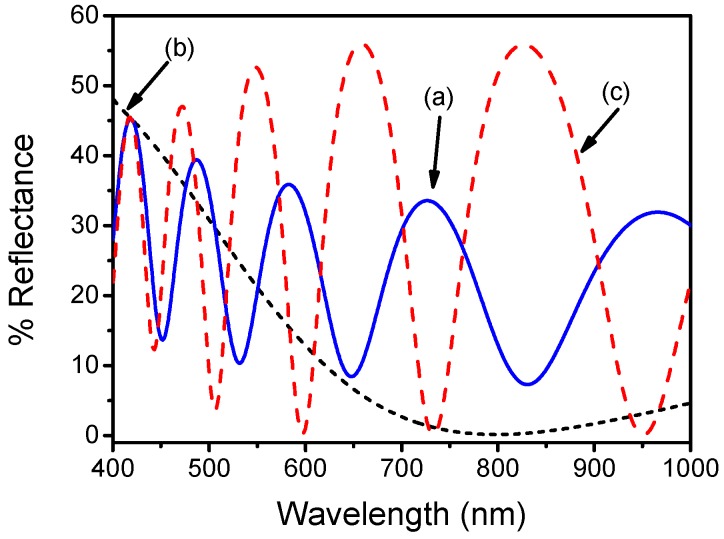
Reflectance spectra obtained in the air from a Si surface with an overlayer of 1000 nm thick SiO_2_ (straight line; a), 100 nm thick Si_3_N_4_ (dotted line, b) or combination of 1000 nm thick SiO_2_ and 100 nm thick Si_3_N_4_ (dashed line, c).

The next steps include further developments regarding the chip cross-section and the fluidic module [39]. It should be noted that any transparent material with refractive index different from the Si substrate could be employed as the interference layer. The dielectric layer should have thickness adequate to produce at least one interference fringe within the spectral range of interest (visible). These materials include SiO_2_ and Si_3_N_4_, but also, materials like TiO_2_, Al_2_O_3_, etc. that can be deposited on the Si wafer. Furthermore, for the wide application of the sensing principle at the Point-of-Care, the biochip should be small and of low-cost. For those reasons, microelectronic processes should be employed, because they guarantee high repeatability, and very high volumes of chips at marginal costs. In this context, the dielectric material that will be employed should be compatible with the microelectronic processes. SiO_2_ and Si_3_N_4_ have the advantage of being compatible with the mainstream microelectronic processing, and can be either thermally grown (SiO_2_) or deposited (Si_3_N_4_) on Si, resulting in surfaces with very low surface roughness, well controlled, and of uniform thickness over large areas and batches. Thus, our study regarding the optimization of chip material was concentrated into these two materials. In particular, SiO_2_ layers with thicknesses ranging from 300 nm to 3000 nm were thermally grown on Si wafers. The lower thickness studied was determined by the fact that smaller thicknesses do not produce interference fringes at VIS/NIR. Respectively, for Si_3_N_4_, the tested layers thicknesses were 100 and 250 nm, since the deposition of thicker layers by low pressure chemical vapor deposition (LPCVD) led to layers with cracks and surface irregularities. In addition, a double layer consisting of a 100 nm thick Si_3_N_4_ layer, deposited on a 1000 nm thick SiO_2_ that was thermally grown onto the Si substrate, was implemented. Typical reflectance spectra recorded for three of the substrates, the 1000 nm thick SiO_2_, the 100 nm thick Si_3_N_4_, and the bilayer of 100 nm thick Si_3_N_4_/1000 nm thick SiO_2_, are provided in Figure 5. In general, it was found that when SiO_2_ was used as interference layer, it provided at least one fringe within the 450–800 nm spectral range for thicknesses over 500 nm. The reflectance spectrum obtained for the Si chip with the combination of Si_3_N_4_/SiO_2_, had the same number of fringes as the 1000 nm thick SiO_2_, but the signal amplitude in minima reached values close to zero reflectance, as in the case of plain Si_3_N_4_ surfaces. For the evaluation of the different chips performance under real assay conditions, a monoclonal antibody against prostate specific antigen (PSA) was immobilized onto the chips, and the responses upon reaction with a calibrator containing 100 ng/mL PSA were compared. It was found that the chips with SiO_2_ layers of thickness ranging from 700 to 3000 nm provided the highest signal during the immunoreaction, as compared to all the other surfaces examined. Especially, for the Si_3_N_4_ or the combined SiO_2_/Si_3_N_4_ overlayer, the occurrence of biomolecular reaction resulted in changes in the amplitude of the interference peak, rather than in spectral shift. Thus, the Si chip with the 1000 nm thick SiO_2_ overlayer was adopted as the optimum substrate.

**Figure 6:**
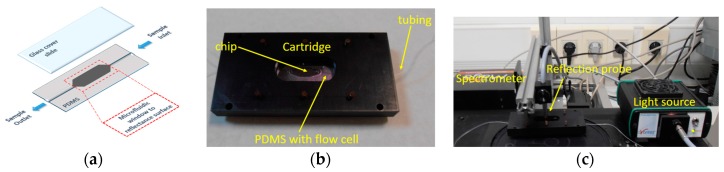
(**a**) Schematic of polydimethylsiloxane (PDMS) gasket with the flow cell and the fluid inlet and outlet. (**b**) Photograph of the cartridge. (**c**) Photograph of the whole experimental set-up.

At the same report, a new cartridge was designed and implemented (Figure 6) so that the chip dimension could be shrunk down to 2 cm × 2 cm. The flow cell was again defined by an opening to a PDMS gasket, to which the inlet and outlet tubing were incorporated. The upper part of the fluidic cell was closed using a standard microscope slide, and the whole structure was placed in the cartridge and secured in place. The fluidic cell thus formed had a volume of 100 μL.

The new cartridge was employed in the immunochemical determination of total- and free-PSA in human serum samples. PSA is considered one of the most reliable markers of prostate cancer, and is used both for the initial disease diagnosis, but most importantly, for monitoring of malignancy recurrence in prostectomized men. There are two forms of PSA in human serum, the free form, which exhibits enzymatic activity as serine protease, and the protein-bound form, that serves as a pool for the active enzyme. Total-PSA serum concentrations (i.e., the sum of free- and protein-bound form) are up to 4 ng/mL in healthy male serum, whereas values over 10 ng/mL are considered as high risk for prostate cancer occurrence. Between these two ranges, there is a “gray zone”, where the elevated PSA concentrations might be due to prostate malignancy or benign hyperplasia. To discriminate the two conditions, the determination of free-PSA to total-PSA ratio, along with total-PSA concentration in serum, are used. To achieve that, highly specific monoclonal antibodies that could recognize only one of the PSA forms were employed for the biofunctionalization of the chip. In addition, in order to achieve detection limits in the ng/mL range, a two-site immunoassay format was employed, which included reaction of the immobilized antibody with the PSA molecules in the calibrators or the samples, followed by binding of a biotinylated anti-PSA antibody, and reaction with streptavidin as a signal enhancement step. Following this procedure, a total-PSA assay with a detection limit of 0.2 ng/mL, and a dynamic range that extended up to 100 ng/mL, was developed. For the free-PSA assay, the detection limit was 0.15 ng/mL, and the dynamic range extended up to 20 ng/mL. Both assays displayed excellent reproducibility (intra-assay coefficient of variation <5.6%) and accuracy (recovery values was ranged from 93 to 108%). Moreover, the same chip could be regenerated and reused for at least 20 times, providing signals in the range of ±5% of the value obtained with the freshly prepared surfaces. The achieved detection limits and working ranges covered the total-PSA assay requirements for determination of the analyte levels in serum blood samples of both healthy and prostectomized individuals. For the free-PSA assay, a lower detection limit would be desirable to cover the free-PSA values in healthy individuals. Nevertheless, free-PSA is needed to be determined only in cases that total-PSA serum values exceed 4 ng/mL, to discriminate between benign prostate hyperplasia and prostate cancer, for which case, the WLRS assay sensitivity is more than adequate. Regarding free- and total-PSA determination by other label-free sensors, there are reports for determination of either analyte based on standard SPR sensors, as well as on localized SPR (LSPR). The WLRS sensor presented significantly lower detection limits compared to standard SPR, which however, was achieved for much longer assay duration [40], whereas its performance is compatible in both terms of assay sensitivity and duration with gold-nanoparticle enhanced SPR [41,42]. On the other hand, LSPR offers higher detection sensitivity compared to WLRS sensor, although the reactions are not monitored in real-time, and the sensors have not been evaluated using human serum samples [43,44]. In addition to good analytical performance, the case of total- and free-PSA determination in the same samples in order to get critical information that could help a patient’s treatment, exposed the need for adaptation of WLRS system to multi-analyte determinations. Besides, another demand that is critical for the diagnostic applications was the shortening of the assay time (70 min for total- or free-PSA), which was pursued mainly by redesign of the fluidic cell.

### 3.3. Multi-Analyte Determinations

**Figure 7:**
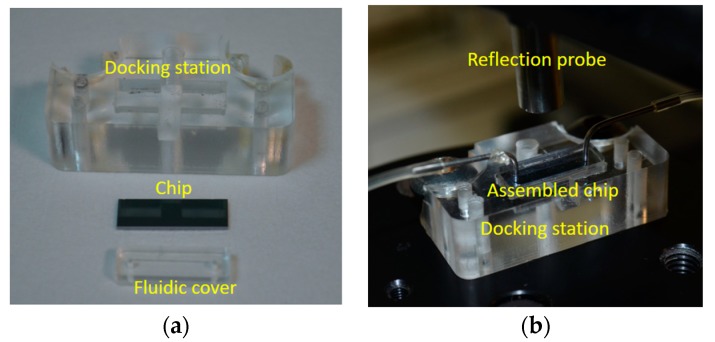
Photograph of the docking station, the chip, and the fluidic cover (**a**) prior to, and (**b**) after their assembly and positioning below the reflection probe.

In order to perform multi-analyte determinations with the WLRS platform, the different recognition molecules were deposited by means of spotting onto spatially discrete areas of the chip, forming the respective reaction zones. To monitor the biomolecular interactions taking place on these reaction zones, the optical set-up was combined with a computer controlled stage, which allowed scanning across the chip. Prior to the realization of this instrument, the fluidic cartridge underwent a radical redesign. At first, the fluidic cell was defined by laser cutting of a 2.0 mm × 12 mm opening on a 200 μm thick double-side adhesive (Tesa Werk Offenburg GmbH, Offenburg, Germany), which was attached to a 2 mm thick PMMA cover, on which the holes for insertion of inlet and outlet tubing have been milled (Figure 7a). The PMMA cover material (PLEXIGLASs GS casted sheets, EVONIK Industries, Essen, Germany) was selected so as to not interfere with the chip probing by the light from the external reflection probe. The volume of the fluidic created upon attaching the described cover to the chip was only 20 μL. To facilitate the multi-analyte determinations by scanning, the assembly with the fluidic cell chip was placed on a specially designed docking station, made by milling of PMMA (Figure 7b). The dimensions of the docking station were 2.0 cm × 3.3 cm, and the recess for positioning of the chip had dimensions marginally bigger than the chip, which now consisted of 5 mm × 15 mm Si wafer dies with a 1000 nm thick SiO_2_ overlayer. The docking station was mounted on a commercially available X–Y stage (Prior Ltd., Cambridge, UK) with position accuracy of 10 μm, in order to allow scanning along the long axis of the chip. The size of the step usually employed was 0.5 mm.

**Figure 8:**
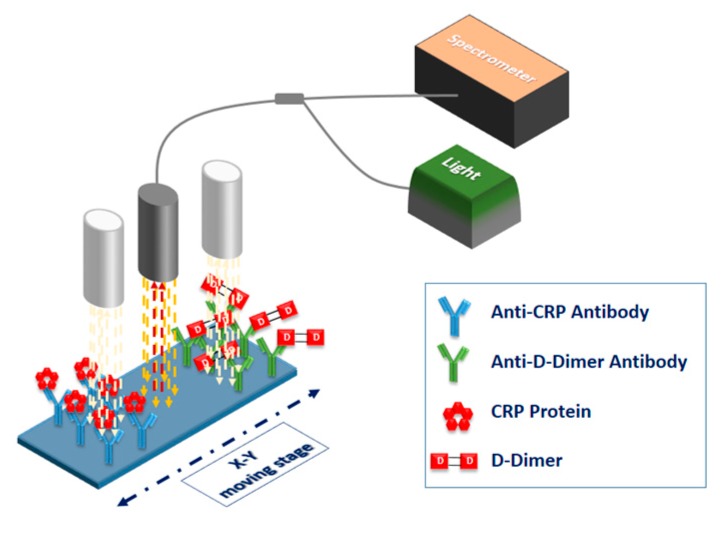
Schematic of the biochip with the two reaction bands and the scanning process.

The particular set-up was applied for the development of a dual-analyte sensor for the simultaneous determination of C-reactive protein (CRP) and D-dimer in human blood plasma [45]. CRP is a biomarker extensively used in clinical practice for diagnosis and monitoring of inflammatory conditions, since in occurrence and persistence of such conditions, its blood levels could be elevated even 1000 times with respect to the values in healthy individuals (from less than 1 μg/μL for normal individuals, to more than 300 μg/mL in acute inflammatory conditions). It has been also proposed as a marker for predisposition to cardiovascular disease when mildly elevated concentrations in blood are detected for long time periods. D-dimer is another marker related to cardiac infarction prognosis, since it is produced by degradation of the fibrin molecules formed when an arterial or venous thrombosis has occurred or is imminent. Increase of its blood plasma concentrations over a threshold value of 0.5 μg/mL, are indicative of an increased risk for thrombosis. Thus, the simultaneous determination of the markers could help to identify individuals at high risk for a myocardial event. To create the dual-analyte biochips, highly specific antibodies against CRP and D-dimer were spotted at spatially distinct areas of the biochip, in order to create two bands with dimensions 1.7 × 3.7 mm^2^, through deposition of 15 × 35 overlapping spots with a pitch of 100 μm, using a solid pin with tip diameter of 375 μm, and employing a standard microarray spotter (Bio-Odyssey Calligrapher, Bio-Rad Laboratories, Hercules, CA, USA). A schematic of the biochip and the scanning process is illustrated in Figure 8.

Three possibilities were investigated: (a) direct detection upon running the calibrators or the samples over the biochip, (b) through a two-site immunoassay, which included reaction with a reporter antibody, and (c) using a biotinylated reporter antibody, followed by reaction with streptavidin. By adopting the optimum reaction times for each of the reaction steps, the detection sensitivity of the two assays could be greatly improved by each additional step. Thus, for CRP, the detection limit dropped from 25 to 2.0, and to 0.05 ng/mL, when detection was performed following the assay format (a), (b), and (c), respectively, whereas the assay duration was increased from 20, to 40, and finally to 45 min, respectively. For D-dimer, direct detection was not possible due to the low signals obtained, but by following format (b) and (c), the detection limit was improved from 200 to 25 ng/mL. Thus, taking into account their relative concentrations in human blood plasma samples from either healthy individuals or patients, for the CRP determination, the direct assay was selected, whereas for D-dimer, the two-site immunoassay with the biotinylated reporter antibody was finally employed. The detection limits achieved following this approach allowed the determination of CRP and D-dimer in blood plasma samples, from both healthy individuals and patients, by adjusting the sample dilution to cover the whole concentration range. As in previous reports, the repeatability (intra-assay CV values from 3.6 to 7.7%; inter-assay CV values from 4.8 to 9.5%, for both assays) and the accuracy of the determinations (% recovery values was ranged from 88.5 to 108% for both assays) was assessed. Thirty-five human blood plasma samples from anonymous donors were analyzed, and the values were found to be in very good agreement with those received for the same samples at a hospital diagnostic laboratory. It should be noted, that compared to other label-free sensors developed for the simultaneous determination of the two-analytes, the sensor developed exceeded in performance either with respect to detection sensitivity or the duration of the assay. There are only two publications regarding the simultaneous determination of CRP and D-dimer with label-free biosensors: the first employing a surface acoustic wave sensor (SAW) complemented with a fluidic that allows detection in sequence of the targeted markers [46], and the second targeting to a Point-of-Care system based on reflectometric interference spectroscopy (RIfS) [47]. Compared to the first system, the WLRS sensor has approximately 5-and 40-times lower quantification limits for CRP and D-dimer, respectively; however, the assay time of the proposed sensor is approximately 3-times longer. Regarding the RIfS sensor, comparison is not possible, due to the lack of extensive analytical data. In addition, neither of these two sensors has been tested with real blood samples. Additional comparison to reports regarding single analyte sensors for either CRP or D-dimer reveals that the developed WLRS sensor has the required detection sensitivity and dynamic range for application in clinical practice. For CRP, in particular, there is a great variety of available assay formats, with sensitivities and working ranges that also vary greatly [48], while for D-dimer, the reported sensors are less abundant, with the electrochemical ones to surpass other types of sensors in analytical performance [49].

The multi-analyte capabilities of the system with regard to the number of analytes that could be determined simultaneously was also accessed by creating several bands of anti-CRP with different width and pitch on a single chip, and comparing the signals obtained from them through scanning to that obtained from a single band biochip. It was found that up to seven bands with width of 0.5 mm and pitch of 1.0 mm could be discriminated using a scanning step of 0.25 mm, since the CV of the values obtained was less than 5%.

### 3.4. Applications to Other Fields

**Figure 9:**
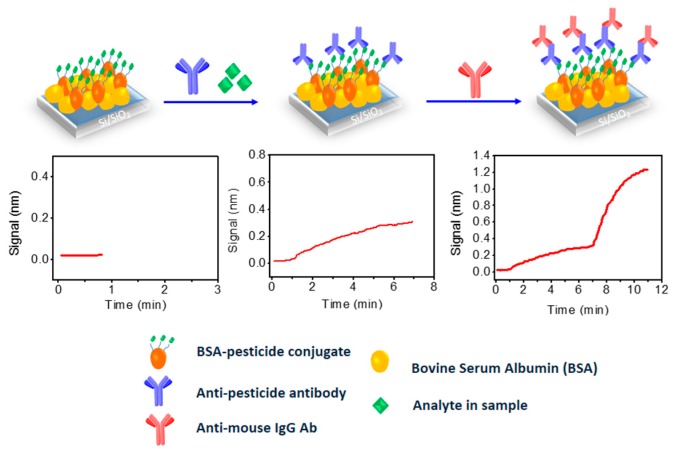
The activation/biofunctionalization procedure of the chip surface and the competitive assay for the determination of pesticides (**top**) along with the expected real-time response graphs (**middle**).

All the applications mentioned so far were related to sensor application in the diagnostic field. Thus, it was really a challenge to investigate the application of WLRS sensing platform to other fields. In this context, the simultaneous detection of three pesticides in water and wine samples was investigated [50]. The three pesticides targeted were chlorpyrifos, thiabendazole and imazalil. Chlorpyrifos is an organophosphate insecticide used to control many different kinds of insects as it acts on their nervous system by inhibiting the action of acetylcholinesterase. It is considered moderately toxic to humans, and it is one of the most widely used organophosphate insecticides in agriculture. Thiabendazole is mainly used as fungicide to control mold formation in fruits and vegetables, but it is also applied as anti-parasitic against worms that infest wild and domestic animals, as well as humans. It is toxic and possibly carcinogenic at high doses. Imazalil is also a fungicide applied both pre- and post-harvest to protect crops; it is toxic and possibly carcinogenic to humans at high doses. For the determination of these substances, a competitive immunoassay format was adopted, which is schematically depicted in Figure 9. According to this, conjugates of each one of the pesticides with a carrier protein (e.g., BSA) were immobilized on spatially discrete areas of a single biochip. For the immunoreaction, a mixture of analyte-specific mouse monoclonal antibodies with the calibrators or the samples was run over the sensing surface, followed by reaction with a secondary polyclonal antibody as signal enhancement step. In fact, the reaction with the secondary antibody lead to a 4-fold signal increase as compared to that obtained by the primary immunoreaction, possibly due to coupling of multiple secondary antibody molecule per immunoadsorbed analyte-specific antibody molecule. Apart from determining the specific signal obtained from each pesticide band upon passing the specific antibody, the possible cross-reaction with the other two pesticides was also investigated. The cross-reactivity was negligible, as it was the non-specific binding signal (signal obtained from the areas between the pesticide–protein conjugates). The assay time required to reach equilibrium at both reaction steps was approximately 50 min (30 min for the immunoreaction of analyte specific-antibody with the immobilized antigen, and 20 min for the immunoreaction of the analyte-specific antibodies with the secondary goat anti-mouse IgG antibody). Nevertheless, the assay time could be reduced down to 10 min, including 6 min for the immunoreaction of analyte specific-antibody with the immobilized antigen, and 4 min for the immunoreaction of the analyte-specific antibodies with the secondary goat anti-mouse IgG antibody. The detection/quantification limits achieved were 30/60 pg/mL for chlorpyrifos and imazalil, and 40/80 pg/mL for thiabendazole, while the working range extended up to 50 ng/mL for chlorpyrifos and imazalil, and to 20 ng/mL for thiabendazole, covering almost three orders of magnitude in pesticide concentration. The limits of quantification were lower than the maximum residual levels (MRLs) for pesticides in drinking water that were set at 100 pg/mL by the EU legislation. Stability and regeneration tests performed using the zero calibrator, showed that for up to 30 assay/regeneration cycles performed over a period of 5 days, the values fall within the mean value ± 2 SD range. Regarding analysis of water and wine samples with the sensor, drinking water could be analyzed without any treatment, while wine samples should be diluted at least 10-times with assay buffer, in order to alleviate any matrix effect. The accuracy of the sensor developed for the simultaneous determination of the three pesticides was evaluated by analyzing 5 wine samples prior to and after addition of known amounts of the three pesticides. The same samples have been analyzed with a validated LC-MS/MS method, and the results obtained with the two methods were in very good agreement. The 10-fold dilution required to alleviate the matrix effect meant that the quantification limits in wines are 10-times higher than those in drinking water (i.e., 0.6 ng/mL for chlorpyrifos and imazalil and 0.80 pg/mL for thiabendazole). Compared to other label-free optical sensors developed for the specific pesticides, the developed immunosensor is about 5-times more sensitive than a single-analyte SPR sensor [51] for chrorpyrifos, and has comparable detection limit with a dual-analyte SPR sensor that included chlorpyrifos and carbaryl [52]. The sensor was also 3-times more sensitive than an SPR sensor for thiabendazole (130 pg/mL) [53]. Detection limits at the same concentration range have been also reported for SPR [54] or optical interferometry sensors [55] employed for the detection of other pesticides. Thus, the WLRS sensor developed could be placed amongst the most sensitive label-free sensors for low-molecular weight contaminants. 

The demonstration of the sensor analytical potential for determination of analytes of low molecular weight in samples other than biological, e.g., environmental or food samples, opens new application fields for the WLRS sensing platform. Multi-analyte determinations are as important in these fields, as in diagnostics. In addition, short analysis time is another prerequisite if on-site application is envisaged.

### 3.5. Current Status and Ongoing Work

In Table 1, the assay and sample type, as well as the analytical characteristics of the assays developed, so far, employing the different versions of the WLPS sensing platform, are listed. Some improvements of the system have already been made, mainly in the direction to build up a more compact and robust system, as well as to improve the performance of some of the already developed assays, and these advancements will be discussed herewith. 

**Figure 10:**
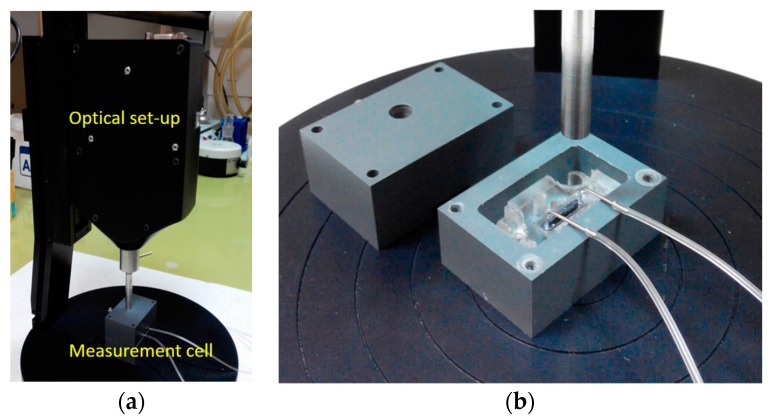
Photographs of the newest version of the WLRS system (**a**) and detailed view of the measurement cell (**b**).

The set-up improvements included the incorporation of all optical components, i.e., the light source, the refection probe, and the spectrometer, into a small size unit (see Figure 10a), and the design and realization of an opaque jacket for the docking station, consisting of two parts (see Figure 10b). The upper part of this jacket had an appropriately drilled hole that allowed the exact positioning of the reflection probe with respect to the biochip. The use of this jacket eliminated all possible interferences from changes of ambient light, making possible the use of the WLRS system in any environment. A small sized peristaltic pump was also tested along with new set-up, indicating the potential for further integration and miniaturization of the whole measurement system.

Regarding the analytical performance of the system, a new assay for PSA was developed that had similar analytical characteristics, but the assay time was reduced from 65 min to 10 min. This significant reduction of the assay time was attributed to the shrinkage of the fluidic cell dimensions (form 100 μL to 20 μL) that allowed for quicker diffusion of the reagents towards the immobilized probe molecules. This fast PSA assay was implemented to determine PSA concentration in forensic cases samples, including swabs and fabrics collected by alleged crime scenes (mainly alleged rape cases) [56]. The results were compared with a semi-quantitative immunochromatographic strip currently used by forensic labs for PSA detection, and the presence or absence of semen was verified by microscopic detection of spermatozoa and male DNA identification through Y chromosome detection. Similarly, a fast assay for CRP was developed employing a two-site immunoassay with biotinylated reporter antibody, and implementation of streptavidin for signal enhancement. The assay could be completed in 10 min and had detection limit of 2.0 ng/mL, which is equal to that obtained with the two-site immunoassay that did not employ a biotinylated reporter antibody and required 40 min to be completed (see Table 1) [57]. The fast assay was evaluated using human blood plasma samples, but also whole blood samples, thus moving the potential of the system for PoC applications one step forward.

## 4. Future Outlook

Throughout the 10 years of the WLRS biosensing system development, a lot of improvements have been made with respect to miniaturization of system and its robustness and analytical performance. At the moment, all the components can be accommodated in a device with a footprint smaller than an A4 page, which could include the electronics for the operation of the different components, as well as the processing of the data. The device is envisioned to be accompanied by cartridges that could incorporate (apart from the sensor and the fluidic cell) all the reagents required for the performance of a particular assay (i.e., buffers, washing solutions, etc.). Depending on the application (diagnostic, environmental or food samples), the cartridge could be destined for single use or multiple uses through regeneration. The design and realization of the disposable cartridge, as well as its production at low cost, is expected to benefit by the widespread application of 3D printing [58]. In addition, the implementation of smartphones as reading or illumination devices to perform optical measurements [59,60,61] paves the way for further miniaturization of optical set-up.

The WLRS approach offer a wide range of characteristics that make it attractive for bioanalytical applications. It is an optical label-free method that provides accurate bioanalytical results in a very short time and at low cost, without any moving optical parts, and without any alignment needs, which is an obstacle for a wide range of other optical techniques. In addition, the use of conventional microelectronic processes, in conjunction with advanced algorithms, could provide multi-analyte determinations by the same measurement set-up. The new approach will target the elimination of any movable parts in the WLRS system through implementation of chips that would have two or more reaction areas that would correspond to different thicknesses of transparent material, e.g., SiO_2_ [62]. These areas will be individually functionalized with different probe molecules, but will be interrogated by a single reflection probe. Through appropriate processing of the spectrum received, the spectra corresponding to the different areas would be extracted, and their shift upon bioreactions would be monitored and correlated to respective analyte concentrations.

The realization of the above-mentioned innovations are expected to further expand the application areas of the WLRS sensing platform, and lead to an instrument with increased potential for commercial exploitation in the near future.

## Figures and Tables

**Table 1:** Overview of the assays developed using the WLRS sensing platform.

Analyte	Assay Format	Assay Duration	Sample	Detection Limit	Dynamic Range	Ref#
C3b	Direct binding/Kinetic	1 min	plasma	20 ng/mL (1/6000 diluted plasma)	0.05–2.0 μg/mL (1/3000–1/100 diluted plasma)	[24]
Total-PSA	Two-site immunoassay with biotinylated reporter antibody and streptavidin	65 min	serum	0.2 ng/mL	0.5–100 ng/mL	
Free-PSA				0.15 ng/mL	0.5–20 ng/mL	[26]
CRP	Direct	20 min	plasma	25 ng/mL	0.05–2.5 μg/mL	
	Two-site immunoassay	40 min		2.0 ng/mL	5.0–1000 ng/mL	
	Two-site immunoassay with biotinylated reporter antibody and streptavidin	45 min		0.05 ng/mL	0.1–10 ng/mL	[32]
D-dimer	Two-site immunoassay	40 min	plasma	200 ng/mL	0.5–10 μg/mL	
	Two-site immunoassay with biotinylated reporter antibody and streptavidin	45 min		25 ng/mL	0.05–1.0 μg/mL	[32]
Chlorpyrifos	Competitive immunoassay	10 min	water or 10-times diluted wine	30 pg/mL	0.06–50 μg/mL	
Imazalil				30 pg/mL	0.06–50 μg/mL	[37]
Thiabendazole				40 pg/mL	0.08–20 μg/mL	
